# Landscape of Peripheral Blood Mononuclear Cells and Soluble Factors in Severe COVID-19 Patients With Pulmonary Fibrosis Development

**DOI:** 10.3389/fimmu.2022.831194

**Published:** 2022-04-26

**Authors:** Zhuolin Wang, Yang Zhang, Rirong Yang, Yujia Wang, Jiapei Guo, Ruya Sun, Yuan Zhou, Li Su, Qing Ge, Yingmei Feng

**Affiliations:** ^1^Department of Immunology, School of Basic Medical Sciences, Peking University. National Health Commission (NHC) Key Laboratory of Medical Immunology (Peking University), Beijing, China; ^2^Beijing Youan Hospital, Capital Medical University, Beijing, China; ^3^Beijing Institute of Hepatology, Beijing Youan Hospital, Capital Medical University, Beijing, China; ^4^Center for Genomic and Personalized Medicine, Guangxi Medical University, Nanning, China; ^5^Department of Immunology, School of Preclinical Medicine, Guangxi Medical University, Guangxi, China; ^6^Department of Biomedical Informatics, Department of Physiology and Pathophysiology, Center for Noncoding RNA Medicine, Ministry of Education (MOE) Key Lab of Cardiovascular Sciences, School of Basic Medical Sciences, Peking University, Beijing, China; ^7^Neuroscience Research Institute, Peking University Center of Medical and Health Analysis, Peking University, Beijing, China; ^8^Department of Integration of Chinese and Western Medicine, School of Basic Medical Sciences, Peking University, Beijing, China

**Keywords:** pulmonary fibrosis, interferon, single-cell RNA sequencing, COVID-19, T cells

## Abstract

Resulting from severe inflammation and cell destruction, COVID-19 patients could develop pulmonary fibrosis (PF), which remains in the convalescent stage. Nevertheless, how immune response participates in the pathogenesis of PF progression is not well defined. To investigate that question, 12 patients with severe COVID-19 were included in the study. Peripheral mononuclear cell (PBMC) samples were collected shortly after their admission and proceeded for single-cell RNA sequencing (scRNA-seq). After 14 days of discharge, the patients were revisited for chest CT scan. PF index (FI) was computed by AI-assisted CT images. Patients were categorized into FI^hi^ and FI^lo^ based on median of FI. By scRNA-seq analysis, our data demonstrated that frequency of CD4+ activated T cells and Treg cells were approximately 3-fold higher in FI^hi^ patients compared with FI^lo^ ones (*p* < 0.034 for all). By dissecting the differentially expressed genes, we found an overall downregulation of IFN-responsive genes (*STAT1, IRF7, ISG15, ISG20, IFIs*, and *IFITMs*) and S100s alarmins (*S100A8, S100A9, S100A12*, etc.) in all T-cell clusters, and cytotoxicity-related genes (*GZMB, PRF1*, and *GNLY*) in CTLs and γδ T cells in the FI^hi^ cohort, compared with FI^lo^ subjects. The GSEA analysis illustrated decreased expression of genes enriched in IFN signaling, innate immune response, adaptive immune response in T cells, NK cells, and monocytes in FI^hi^ patients compared with FI^lo^ ones. In conclusion, these data indicated that the attenuated IFN-responsive genes and their related signaling pathways could be critical for PF progression in COVID-19 patients.

## Introduction

The coronavirus disease (COVID-19) pandemic has resulted in substantial morbidity and mortality worldwide (1). COVID-19 affects multiple tissues and systems, with the lungs being the primary source of infection and injury. Despite the fast progress in early diagnosis, disease management, and vaccine development, the acute and chronic pulmonary consequences, including pneumonia, acute respiratory distress syndrome (ARDS), and pulmonary fibrosis (PF), remain major concerns in patients with SARS-CoV-2 infection (2). In particular, a substantial percentage of post-COVID-19 survivors have evidence of PF, such as long-lasting lung functional impairment and radiologic abnormalities (persistent ground-glass opacities, nodules, consolidation, or even reticular abnormalities, traction bronchiectasis, and honeycomb cyst) (3–11).

The prevalence of PF is directly correlated with initial disease severity (2). It remains unclear why most patients recover from the inflammatory damage while a proportion develop excessive fibrosis with loss of quality of life and pulmonary dysfunction (12). Many local factors have been found to be closely associated with PF, such as direct SARS-CoV-2-mediated alveolar and airway epithelial cell death (13, 14); crosstalk between the alveolar epithelium, endothelium, and fibroblasts (15); complement deposition and micro-thrombosis in pulmonary capillaries (16); and pulmonary cellular senescence (17). The induction of oxidative stress, excessive production of reactive oxygen species, and the presence of high levels of fibrotic factors including TGF-β, FGF, and PDGF in the lung also contribute to PF (18, 19).

At the cellular level, increased neutrophil, eosinophil, megakaryocyte, and erythroid cell frequencies (20–26); decreased lymphocytes, natural killer (NK) cells, and plasmacytoid dendritic cells (pDCs) (20, 27–29); the appearance of dysfunctional classical monocytes with upregulated S100 alarmins and downregulated MHC class II expression; and loss of non-classical monocytes were associated with severe COVID-19 (20, 24, 30–34). CD4^+^, but not CD8^+^ T cells or B cells, were found to have strong association with COVID-19 severity, with rapid induction of SARS-CoV-2-specific CD4^+^ T cells being associated with mild disease and accelerated viral clearance, while delayed or absence of SARS-CoV-2-specific CD4^+^ T cells being associated with severe or fatal COVID-19 (35, 36). Nevertheless, limited studies have been performed to understand different degrees of PF present in convalescent COVID-19 patients.

To this end, we examined the circulating soluble factors and single-cell RNA sequencing (scRNA-seq) of peripheral blood mononuclear cells (PBMCs) in 12 severe COVID-19 patients upon admission. We then analyzed the association of these variables with PF index obtained from their chest CT imaging within 14 days of discharge.

## Materials and Methods

### Study Design and Participants

A total of 12 patients with severe COVID-19 who were hospitalized in Beijing Youan Hospital from January 24 to May 18, 2020, were included in this study. Diagnosis of COVID-19 was confirmed by RT-qPCR using the SARS-CoV-2 RNA detection kit (BioGerm Medical Biotechnology Co., Ltd., Shanghai, China) of pharyngeal swabs on the ABI 7500 Real-Time PCR System according to the World Health Organization (WHO) guidelines. The severity of COVID-19 was defined following a 7th version of the instruction of the National Institute for Viral Disease Control and Prevention, which included clinical features, laboratory measurements, and CT scans. General information including age, disease history, and drug use were recorded. Hypertension was defined as systolic blood pressure ≥140 mmHg or diastolic blood pressure ≥90 mmHg or use of antihypertensive drug. Diabetes was defined as a fasting blood glucose level ≥7.0 mmol/L or use of antidiabetic drugs.

The study has been reviewed and approved by the Institutional Review Board of the Capital Medical University prior to conducting clinical trials. All participants provided written informed consent before they enrolled in the study.

### AI-Assisted Quantification of Pulmonary Fibrosis

After discharge, 12 patients were revisited for general examination. The extent of PF was assessed by AI-Based CT Imaging System (Dr. Wise @Pneumonia, version 1.0, Beijing Deepwise & League of Ph.D. Technology Co., Ltd., China) and have been previously described (37). Fibrosis volume or the percentage within the entire lung was used to determine the presence and severity of PF (fibrosis index, FI).

### Biochemical Measurement

Venous blood samples were collected upon admission to measure the red blood cell count, white blood cell count and differential white blood cell count, serum creatinine, alanine aminotransferase, aspartate aminotransferase, total bilirubin, creatinine kinase, myoglobin, and C-reactive protein after overnight fasting.

### Cytokine and Chemokine Measurement

To explore where any cytokines and chemokines were related to PF development, fasting plasma samples were collected shortly after admission. The serum cytokine and chemokine profiles of the 12 patients were determined using a convenient Bioplex Kit Assay (LINCO Research, Inc., USA) according to the manufacturer’s instruction.

### PBMC Isolation and scRNA-seq Library Preparation

Peripheral blood mononuclear cells (PBMCs) were prepared from whole blood using Ficoll-Paque density gradient centrifugation (HISTOPAQUE^®^ 1083, Sigma, USA) according to the manufacturer’s instructions. After centrifugation, the opaque interface containing the mononuclear cells was transferred into a 15-ml conical centrifuge tube and washed 3 times with 10 ml of PBS. Single-cell suspension (1,000 cells/µl, Cell viability >85%) was loaded into a Chromium controller (10X Genomics, USA) to generate single-cell Gel Beads-In-Emulsions (GEMs). The scRNA-seq library preparation was performed with chromium single cell 3’ reagent kit v2 (10x Genomics, USA) following the instruction.

### scRNA-seq Data Processing and Quality Control

Raw FASTQ sequencing files were aligned against the Human GRCh38 genome and processed through Cell Ranger (v3.1.0) following the official pipeline. Moreover, COVID-19 genome (NC_045512, https://www.ncbi.nlm.nih.gov/sars-cov-2) was converted into standard reference and used as input for Cell Ranger alignment. The expression matrixes of 12 samples were loaded into R (v4.0.3), integrated together and analyzed by the Seurat (v4.0.3) package (38). Samples that meet the following criteria were selected for subsequent analysis: (1) cells that expressed >200 and <2,500 genes; (2) genes that expressed >20 cells; and (3) the percentage of mitochondrial gene <15. The harmony (v0.1.0) package was used to reduce the batch effect. After the quality control, there were 166,265 cells and 16,436 genes for downstream analysis.

### Clustering and Cell-Type Annotation

After normalizing and scaling, the “RunPCA” function in Seurat was used for Dimension reduction. Next, we selected the first 40 PCs and resolution 1.5 for clustering, finally leading to 40 clusters. The “RunUMAP” function was performed to obtain the spatial visualization of each cell. The differentially expressed genes (DEGs) of each cell cluster was calculated by the “FindAllMarkers” function. Based on the cluster-specific biomarkers (**Figure 2C**), the clusters were annotated as 11 transcriptionally distinct clusters. For further detailed analysis, T cell, NK cell, Monocyte, B cell, pDC, and cDC were separately reclustered and annotated.

### DEGs Identification and Pathway Enrichment

The DEGs between FI^hi^ and FI^lo^ groups were identified with theDESeq2 (v1.30.1) package (39). Based on the canonical pathways, which are described in the MSigDB database, “GSEA” in GSEABase (v1.54.0) package was applied to assess the pathway activity in FI^hi^ and FI^lo^ groups (40).

### TCR Sequencing and Analysis

Full-length TCR V(D)J repertoires were sequenced by Chromium Single Cell Immune Profiling (10X Genomics). Assembling and paired clonotype calling of TCR libraries were processed by Cell Ranger (v3.1.0) and the GRCh38 VDJ reference file was provided by Cell Ranger (refdata-cellranger-vdj-GRCh38-alts-ensembl-4.0.0). Next, the TCR repertoire matrixes were loaded into R and processed by scRepertoire (v1.3.4) package. TCR that detected on non-T cell was filtered out. In order to categorize the frequency of clone types, it was divided into 6 levels [Hyperexpanded (100 < X ≤ 500), Large (20 < X ≤ 100), Medium (5 < X ≤ 20), Small (1 < X ≤ 5), Single (0 < X ≤ 1), and NA]. The “combinExpression” function was used to attach the clonotypic information to the Seurat object. The diversity of TCR was evaluated by Shannon and Abundance-based Coverage Estimator (ACE).

### SCENIC Analysis

Regulatory network analysis was performed by the SCENIC (v1.2.4) package using the default parameters (41). The co-expression modules were based on cisTarget databases (hg38:refseq-r80:500bp_up_and_100bp_down_tss, hg38:refseq-r80:10kb_up_ and_down_tss). To save the computational source, 10,000 T cells were randomly selected and used as input for the network inference step. The AUCell scores were then calculated using all 53,488 T cells (42).

### Pseudotime Transcriptional Trajectory Analysis

A total of 30,000 T cells were randomly selected from clusters annotated as CD4^+^ T cell, CD8^+^ T cell, Treg, and cycling T cell (the cluster T13 was not included as the majority of the cells in this cluster were from patient #02). Their RNA counts were used as the input for Monocle (v2.18.0) for downstream analysis. “FindAllMarkers” was used to find the differential expression and the genes with adjusted *p*-value < 0.01 were selected for further analysis. The DDRTree method was used for Dimension reduction.

### Cell–Cell Interaction

CellPhoneDB (v2.1.7) was utilized in the analysis of cell–cell interaction (43). The expression matrixes of FI^hi^ and FI^lo^ were separately input into CellPhoneDB, and possible biological interactions between any two cell types were evaluated.

### Statistics

All statistical analyses were performed using SPSS data analysis statistics software system version 21.0 (SPSS Inc., Chicago, IL, USA). The baseline characteristics were expressed as median with interquartile range (IQR). Continuous data were compared by the Student’s *t*-test or Mann–Whitney test, while categorical data were assessed using Fisher exact test. The correlations of clinical measures, cytokine levels, and the transcriptome features of PBMC cell clusters were determined by Pearson or Spearman correlation coefficients based on normal or non-normal distribution between variables and visualized using the corrplot package (v0.89) (44).

## Results

### General Characterization of Convalescent COVID-19 Patients With PF

To determine whether the cellular and soluble factors collected upon admission correlated with the severity of pulmonary fibrosis at discharge, 12 patients with severe COVID-19 admitted in Beijing Youan Hospital were recruited. Fourteen days after discharge, they were revisited for chest CT scan. Fibrosis index (FI), or the percentage of fibrosis volume in the entire lung was computed from the AI-based chest CT imaging system (37, 45). Based on the median value (0.0567) of FI, 12 COVID-19 patients were divided into two groups. The demographic information and clinical features of 12 patients are shown in **Table 1**. Compared with FI^lo^ patients, serum total bilirubin levels had a 2.1-fold increase in FI^hi^ patients [7.3 (5.5-8.6) μmol/L vs. 15.6 (11.9-23.9) μmol/L, *n* = 6 for each, *p* = 0.015] (**Figure S1A**). Quantified by ELISA, circulating levels of EGF, FGF-2, IL-2, IL-3, IL-4, and IL-7 were 6.1-, 1.6-, 4.3-, 2.4-, 3.3-, and 3.5-fold lower than that of FI^lo^ patients, respectively (*p* < 0.03 for all) (**Table 2** and **Figures S1B**–**G**).

**Table 1:** General characteristics of COVID-119 patients classified by pulmonary fibrosis index.

	All patients	COVID-19 patients		*p-*value
		FI ≤ 0.0567	FI > 0.0567	
Number	12	6	6	
Collection days since disease onset (days)	13.0 (5.5–18.5)	13.0 (8.5–19.5)	14 (2.75–23.5)	0.94
Male (%)	8 (66.7%)	4 (66.7%)	4 (66.7%)	1.00
Age (years)	63 (47–70)	49 (44–69)	70 (61–72)	0.067
Hypertension (%)	2 (16.7%)	1 (16.7%)	1 (16.7%)	1.00
Diabetes (%)	0 (0%)	0 (0.0%)	0 (0.0%)	1.00
Cardiovascular disease (%)	3 (25.0%)	1 (16.7%)	2 (33.0%)	1.00
Body temperature on admission (°C)	37.1 (36.6–38.0)	37.2 (36.8–38.0)	37.1 (36.5–38.1)	0.97
SpO_2_ (%)	96.9 (93.3–99.4)	98.3 (93.3–99.5)	95.5 (92.0–99.3)	0.82
Red blood cell count (×10^12^/L)	4.34 (4.18–4.92)	4.49 (3.93–4.95)	4.30 (4.21–4.96)	1.00
White cell count (×10^9^/L)	6.69 (4.58–7.34)	5.84 (4.32–7.90)	6.88 (4.90–11.53)	0.59
Neutrophil percentage (%)	71.9 (63.5–80.3)	66.8 (57.3–78.6)	76.4 (67.1–83.2)	0.29
Lymphocyte percentage (%)	19.9 (12.6–24.6)	21.8 (13.1–29.4)	16.7 (10.5–24.6)	0.24
Monocyte percentage (%)	5.5 (5.0–7.0)	5.9 (5.3–8.3)	5.1 (3.2–6.7)	0.24
Platelet count (×10^9^/L)	189 (125–283)	203 (169–348)	140 (107–257)	0.31
Serum creatinine (μmol/L)	63 (47–76)	73 (45–78)	54 (47–75)	0.47
Alanine aminotransferase (U/L)	35 (28–79)	30 (28–83)	42 (26–78)	1.00
Aspartate aminotransferase (U/L)	39 (24–75)	26 (20–83)	53 (32–77)	0.39
Total bilirubin (μmol/L)	8.9 (6.7–16.1)	7.3 (5.5–8.6)	15.6 (11.9–23.4)	0.015
Creatine kinase (U/L)	68 (55–96)	68.5 (52.0–85.3)	65.0 (48.8–230.0)	0.97
Myoglobin (ng/ml)	63 (45–133)	58 (40–67)	108 (42–285)	0.39
C-reactive protein (ng/ml)	35.9 (14.0–50.7)	19.7 (7.8–41.2)	44.2 (30.5–55.8)	0.093

FI, pulmonary fibrosis index; computed by AI-assisted CT imaging. Data are expressed as median with interquartile range (IQR). Continuous data were compared by the Student’s t-test or Mann–Whitney test. Categorical data were assessed using Fisher exact test.

**Table 2:** Serum soluble factors and cytokines in patients with severe COVID-19 when categorized by pulmonary fibrosis index.

Cytokines median, pg/ml (IQR)	FI^lo^(pg/ml, *n* = 5)	FI^hi^(pg/ml, *n* = 6)	*p*-value
sCD40L (pg/ml)	3,402.00 (2,325.00–7,549.00)	922.75 (359.63–2,169.00)	0.09
EGF (pg/ml)	164.82 (69.24–174.67)	27.02 (5.19–73.45)	0.017
Eotaxin (pg/ml)	102.87 (70.00–245.08)	182.33 (61.79–265.31)	0.79
FGF-2 (pg/ml)	273.92 (222.58–416.48)	168.85 (123.22– 188.45)	0.004
FLT-3L (pg/ml)	17.75 (14.2–28.24)	9.84 (8.35 –17.29)	0.08
Fractalkine (pg/ml)	205.75 (46.82–268.60)	47.29 (17.40–192.96)	0.25
G-CSF (pg/ml)	31.61 (11.73–37.27)	90.04 (31.68–108.94)	0.08
GM-CSF (pg/ml)	14.31 (2.52–53.33)	13.73 (3.31–91.75)	1.00
GRO-alpha (pg/ml)	21.13 (9.75–25.93)	6.66 (1.54–25.19)	0.25
IFN-alpha2 (pg/ml)	67.61 (43.46–130.94)	63.40 (45.01–108.10)	1.00
IFN-gamma (pg/ml)	12.94 (4.11–28.68)	3.26 (0.80–13.93)	0.18
IL-1 alpha (pg/ml)	25.48 (6.01–50.77)	15.23 (6.09–35.68)	0.43
IL-1 beta (pg/ml)	12.58 (8.29– 20.93)	7.04 (2.37–12.01)	0.13
IL-1RA (pg/ml)	28.75 (12.97–38.34)	191.62 (20.41– 710.77)	0.18
IL-2 (pg/ml)	1.24 (0.61–2.92)	0.29 (0.18–0.45)	0.009
IL-3 (pg/ml)	4.76 (3.78–12.22)	1.95 (1.15–3.94)	0.030
IL-4 (pg/ml)	2.00 (1.31–4.94)	0.60 (0.37–0.98)	0.017
IL-5 (pg/ml)	10.91 (5.31–19.62)	7.77 (3.31–10.50)	0.18
IL-6 (pg/ml)	10.58(2.39-16.72)	36.04(3.03-45.15)	0.25
IL-7 (pg/ml)	5.89 (4.35–8.77)	1.69 (0.63–2.68)	0.004
IL-8 (pg/ml)	4.95(4.09-7.02)	1.80(0.88-7.92)	0.33
IL-9 (pg/ml)	23.04 (15.22–31.07)	19.51 (13.81–22.19)	0.25
IL-10 (pg/ml)	20.08 (10.48–25.89)	36.37 (12.04–43.88)	0.25
IL-12 (p40) (pg/ml)	94.36 (61.97–177.39)	76.20 (53.36–132.35)	0.79
IL-12 (p70) (pg/ml)	15.80 (8.63–31.42)	3.56 (1.95–9.39)	0.052
IL-13 (pg/ml)	25.17 (14.48–45.14)	9.48 (5.33–19.62)	0.052
IL-15 (pg/ml)	14.81 (9.97–33.52)	15.56 (3.21–27.82)	0.66
IL-17A (pg/ml)	8.23 (4.00–14.95)	4.19 (2.00–10.72)	0.66
IL-17E/IL-25 (pg/ml)	547.44 (363.22–1,541.50)	449.48 (227.01–514.68)	0.25
IL-17F (pg/ml)	24.34 (13.82–97.10)	16.61 (12.96–20.16)	0.25
IL-18 (pg/ml)	40.57 (23.15–82.14)	47.28 (9.76–101.11)	1.00
IL-22 (pg/ml)	29.02 (12.21–72.66)	12.67 (5.76–26.29)	0.33
IL-27 (pg/ml)	3,185.00 (1,972.00–5,375.50)	4,731.50 (2,041.86–9,794.50)	0.66
IP-10 (pg/ml)	516.01 (286.87–5,415.50)	1,430.89 (461.52–6,396.00)	0.66
MCP-1 (pg/ml)	312.60 (200.83–420.02)	445.17 (243.71–1,207.82)	0.33
MCP-3 (pg/ml)	79.87 (35.49–96.79)	48.34 (30.83–85.04)	0.66
M-CSF (pg/ml)	166.29 (105.13–351.51)	149.12 (103.09–305.66)	0.66
MDC (pg/ml)	264.15 (205.57–757.69)	292.32 (231.34–676.05)	0.79
MIG (pg/ml)	3,397.00 (2,141.00–6,438.00)	3,080.69 (201.68–10,622.25)	0.79
MIP-1alpha (pg/ml)	42.66 (37.02 – 77.46)	30.02 (26.85 – 44.09)	0.052
MIP-1beta (pg/ml)	42.86 (33.01 – 84.64)	142.37 (59.07 – 214.37)	0.082
PDGF-AA (pg/ml)	4,375.00 (1,711.00–7,771.00)	881.88 (602.08–2,175.25)	0.052
PDGF-AB/BB (pg/ml)	23,924.00 (19,967.50–32,025.00)	18,641.50 (10,617.50–25,822.00)	0.18
RANTES (pg/ml)	3,804.00 (3,579.00–4,517.00)	4,210.00 (4,039.25–6,304.25)	0.18
TGF-alpha (pg/ml)	10.32 (6.63–14.27)	4.64 (4.06–9.14)	0.13
TNF-alpha (pg/ml)	64.45(55.93-114.86)	109.23(39.07-142.36)	0.79
TNF-beta (pg/ml)	10.40 (3.76–21.08)	13.72 (1.89–145.71)	0.93
VEGF-A (pg/ml)	255.19 (195.51–319.61)	366.98 (189.49–1,392.16)	0.79

FI, pulmonary fibrosis index. FI^lo^ and FI^hi^ were categorized by FI ≤ 0.057 or FI > 0.057, respectively.

### Dysregulated T-Cell Differentiation in COVID-19 Patients With High PF Index

**Figure 1:**
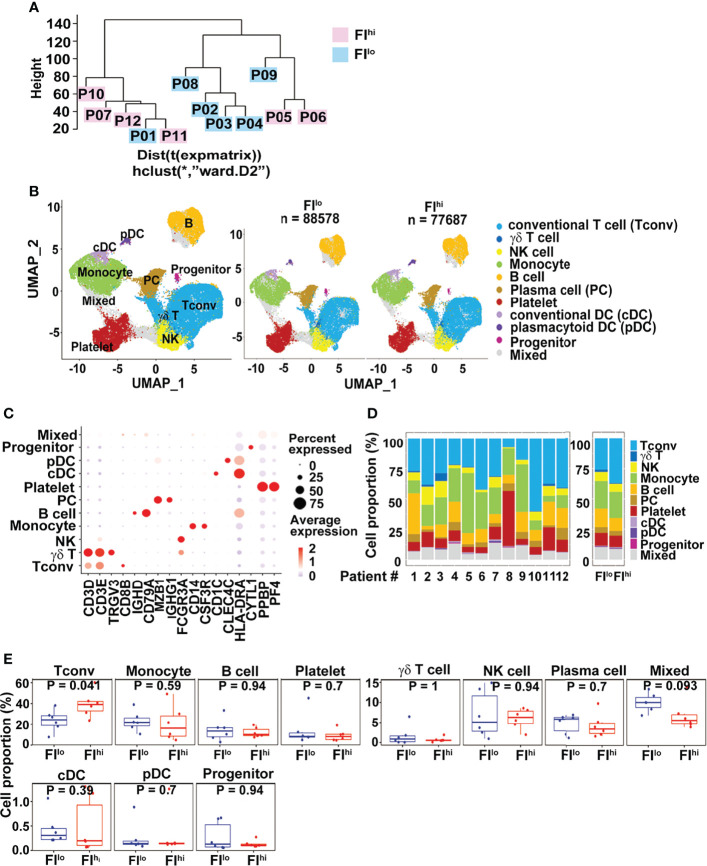
Increased CD3^+^ conventional T cells in FI^hi^ patients with severe COVID-19. **(A)** Pseudo-bulk analysis and clustering of the samples collected from 12 patients with severe COVID-19. The patients were evaluated by chest CT imaging system at discharge, and FI values were given. Based on the median value (0.0567) of FI, the patients were divided into two groups, FI^lo^ (P01-04, P08-09) and FI^hi^ (P05-07, P10-12). **(B)** UMAP visualization of PBMC clusters. **(C)**. Dot plot showing the row-scaled expression of selected signature genes for each cluster. **(D)** Cell composition varied among all patients studied. **(E)**. Increased conventional T-cell proportion in FI^hi^ cohorts.

To compare the transcriptome features of PBMCs between FI^lo and^ FI^hi^ patients, we performed the scRNA-seq using PBMCs isolated from the patients. All PBMC samples were collected 13.0 (IQR, 5.5–18.5) days since disease onset. By pseudo-bulk expression-based hierarchical clustering, the transcriptomic features of PBMCs in some of the patients with high FI (P07, P10-12) were clustered together. However, the FI^hi^ (P05-P07, P10-12) and FI^lo^ (P01-04, P08-09) patient cohorts were not separated well from each other (**Figure 1A**).

We applied cluster-specific biomarkers to resolve cell types and functional states into 11 transcriptionally distinct clusters (**Figures 1B, C**). The biomarkers used for cell population definition included CD3D, CD3E, TRGV3, CD8B, IGHD, CD79A, MZB1, IGHG1, FCGR3A, CD14, CSF3R, CD1C, CLEC4C, HLA-DRA, CYTL1, PPBP, and PF4. Each cell population was identified by the biomarkers as follows: *CD3D*^+^ and *CD3E*^+^ for conventional T cells (Tconv); *CD3D*^+^, *CD3E*^+^, and *TRGV3*^+^ for γδT cells; *FCGR3A*^+^ for NK cells; *CD14*^+^ and *CFS3R*^+^ for monocytes; *IGHD*^+^ and *CD79A*^+^ for B cells; *MZB1*^+^ and *IGHG1*^+^ for plasma cells; *PPBP*^+^ and *PF4*^+^ for platelets; *CD1C*^+^ and *HLA-DRA*^+^ for conventional DC (cDC); *CLEC4C*^+^ and *HLA-DRA*^+^ for plasmacytoid DC (pDC); and *CYTL1*^+^ for progenitor cells. The proportions of each cluster varied among patients (**Figure 1D**). Compared to FI^lo^ individuals, the FI^hi^ ones had significantly higher percentage of Tconv cells (**Figure 1E**). By contrast, the proportions of other cell types, including γδ T cells, B cells, NK cells, and myeloid cells, were comparable between two groups (*p* ≥ 0.09 for all) (**Figure 1E**).

**Figure 2:**
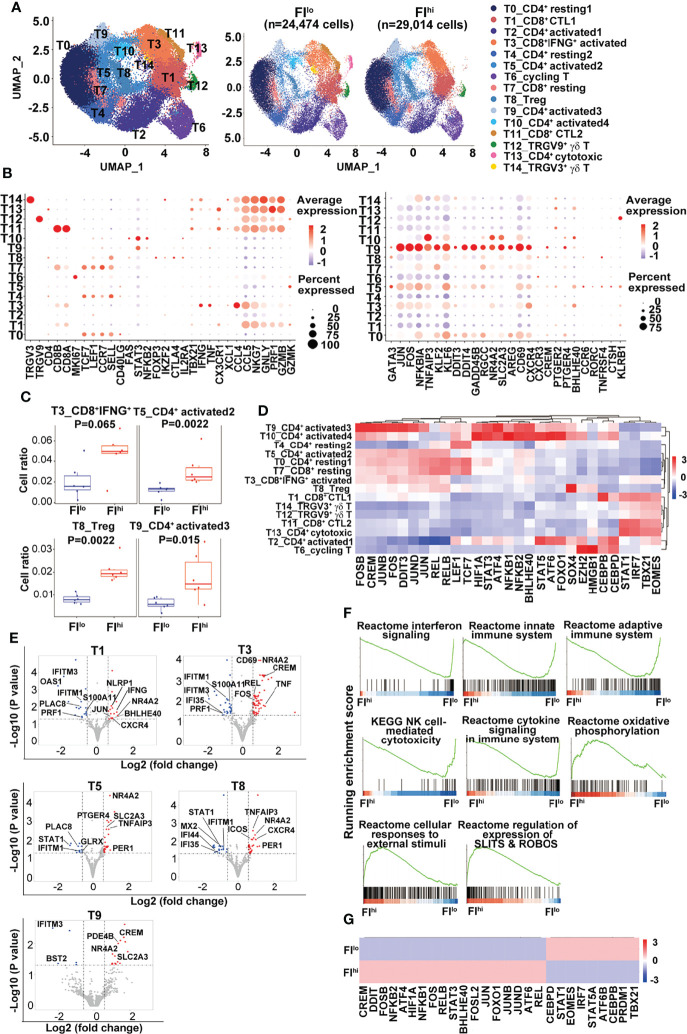
Increased percentage of activated CD4^+^ T cells and Treg cells in FI^hi^ patients with severe COVID-19. **(A)** UMAP visualization of T-cell clusters. **(B)** Dot plot showing the row-scaled expression of selected signature genes for each cluster. **(C)** Comparison of the proportions of T-cell clusters between FI ^lo^ and FI^hi^ COVID patients. **(D)** Heatmap of the transcription factors of each cluster, with mean area under the curve (AUC) scores are shown. **(E)** Volcano plot showing DEGs (*p*-values < 0.05) in the comparison of each T-cell cluster derived from FI^lo^ and FI^hi^ patients. **(F)** GSEA analysis of differentially expressed genes (DEGs) in T cells in the comparison of FI^lo^ and FI^hi^ patients with severe COVID-19. **(G)** Heatmap comparison of the transcription factors in T cells derived from FI^lo^ and FI^hi^ patients.

As some of the T cell-related cytokines were reduced and Tconv ratios were increased in the patients with FI^hi^, we characterized T cells in more detail and identified fifteen clusters, including eight CD4^+^, four CD8^+^ T-cell clusters, one cycling T-cell cluster (T6), and two γδ T-cell clusters (T12 *TRGV9*^+^ and T14 *TRGV3*^+^), based on the selected marker genes (**Figures 2A, B** and **Figure S2A**).

Compared to FI^lo^ patients, the FI^hi^ individuals had significantly higher percentages of *CD4^+^* activated T cells (T5 and T9) and Treg cells (T8) [T5: 0.013 (0.009–0.015) vs. 0.024 (0.021–0.042), *p* = 0.002; T9: 0.006 (0.004–0.009) vs. 0.014 (0.011–0.029), *p* = 0.015; T8: 0.008 (0.006–0.010) vs. 0.019 (0.018–0.024), *p* = 0.002; *n* = 6 for each group] (**Figure 2C**). A mild expansion of *CD8^+^IFNG*^hi^ activated T cells (T3) was also observed in FI^hi^ patients [0.016 (0.009–0.034) vs. 0.049 (0.038–0.060), *p* = 0.065, *n* = 6 for each group] (**Figure 2C**), except that the other cell populations did not differ between two groups (*p* ≥ 0.16).

### Defective IFN Responsiveness in T Cells of COVID-19 Patients With High PF Index

Next, the transcription factors (TFs) with high activity within each cluster were revealed by TF regulatory network analysis (46) (**Figure 2D**). The clusters with resting T-cell features (*LEF1*, *TCF7*) correspond to naïve or central memory T cells including T0 (*CD4*^+^ resting1), T4 (*CD4*^+^ resting2), and T7 (*CD8*^+^ resting). We observed one cluster of *CD8^+^IFNG*^hi^ activated T cells (T3) and two clusters of CD8^+^ cytotoxic T (T1 and T11, CTL1-2) cells expressing different combinations or levels of cytotoxicity-related genes. The rest of CD4^+^ T cells were divided into one cluster (T8) of Treg cells with *FOXP3* expression; four clusters of CD4^+^ activated T cells (T2, T5, T9, and T10, activated 1–4); and one cluster of CD4^+^ cytotoxic T cells (T13). Single-cell TCR sequencing revealed clonal expansion in CD8^+^ activated T cells, CTLs, and CD4^+^ cytotoxic T cells (**Figure S2B**). The majority of cells in cluster T13 from the patient P02 contained highly expanded oligoclonal T cells and thus excluded in the later analysis. The pseudo-time analysis further showed a V-shaped trajectory path with resting T-cell clusters (T0, T4, and T7) being mostly at the left side while the CD8^+^ T cells with high levels of cytolytic gene expression (T1 and T11) being mostly at the right side of the path (**Figure S2C**). No difference was found in TCR diversity between FI^hi^ and FI^lo^ cohorts (**Figure S2D**).

Notably, CD4^+^ T cells in cluster T9 expressed high levels of *KLF6* that positively regulates the expression of TGFβ and molecules in the TGFβ pathway (47), *AREG* (amphiregulin) that is involved in pulmonary fibrosis (48), and *CXCR4* that may promote T-cell migration to the lung (49). A group of TFs with high activity were also revealed in cluster T9, including AP-1, NF-κB, CREM, BHLHE40, STAT3, and stress-response sensors HIF1α, ATF4, and DDIT3. BHLHE40 is a stress-responsive TF that promotes inflammatory Th1 cell differentiation while CREM is a cAMP-dependent TF that promotes oxidative phosphorylation, STAT3 expression, and IL-17 expression (50–52). These transcriptome features indicate that the cells in cluster T9 undergo high level of endoplasmic reticulum (ER) stress that may lead to dysregulated effector T-cell survival and/or differentiation (53–55).

By DEGs (56), we found an overall downregulation of IFN-responsive genes (*STAT1, IFIs, IFITMs*, and *OASs*) and upregulation of cell exhaustion-related nuclear receptor *NR4A2* in all the T clusters in the FI^hi^ cohort, when compared with FI^lo^ subjects (**Figure 2E** and **Table S1**). CTLs in T1 and T3 clusters in FI^hi^ cohorts also showed downregulation of cytotoxicity-related gene *PRF1* and upregulation of cytokines (*IFNG* and *TNF*) and transcription factors (*BHLHE40* and *CREM*) that promote inflammatory cytokine production. The activated CD4^+^ T cells in clusters T5 and T9 showed higher expression of glucose transporter *SLC2A3* in the FI^hi^ group. The GSEA analysis showed that FI^hi^ patient-derived total T cells or T cells in various clusters had decreased expression of genes enriched in IFN signaling, innate immune response, adaptive immune response, and NK cell-mediated cytotoxicity (**Figure 2F** and data not shown). The increased expression of genes in FI^hi^-derived effector T cells, in particular, cells in clusters T3, T5, and T11, were enriched in cellular responses to external stimuli, regulation of expression of SLITS and ROBOS, and oxidative phosphorylation (**Figure 2F**).

We further used TF regulatory network analysis to compare the two patient cohorts and found that the FI^hi^ one had lower activities of IRF7/STAT1 and TBX21/EOMES that promote antiviral immune responses but higher activities of AP-1, NF-κB, stress-related ATF4/6, DDIT3, HIF1α, CREM, and BHLHE40 that likely mediate inflammatory cytokine production and immunopathology (57) (**Figure 2G**).

Together, these data indicate that the patients with high PF index had dysregulated T-cell differentiation with defective IFN responsiveness and cytotoxicity, and expansion of inflammation-promoting CD4^+^ T cells.

### Defective Antiviral Response in NK, Monocytes, and Dendritic Cells of Severe COVID-19 Patients With High PF Index

**Figure 3:**
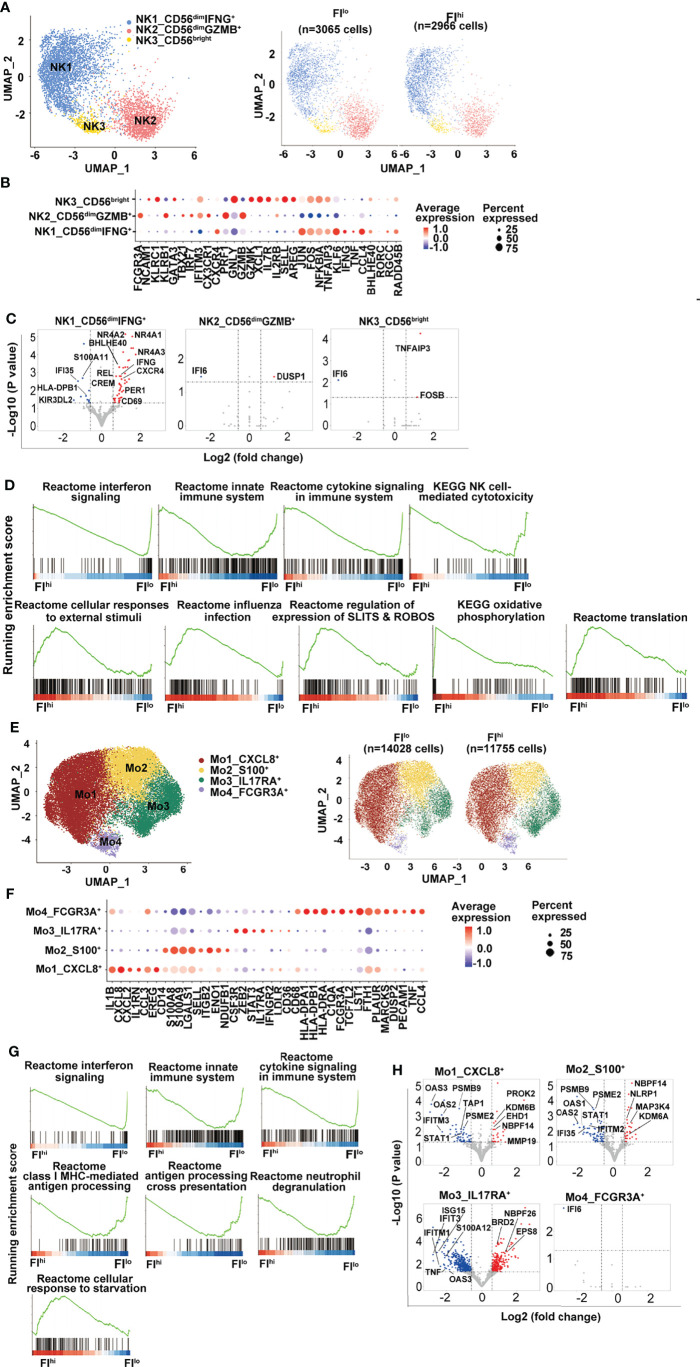
Attenuated global antiviral response in FI^hi^ patients with severe COVID-19. **(A)** UMAP visualization of NK cell clusters. **(B)** Heatmap showing the row-scaled expression of selected signature genes for each cluster. **(C)** Volcano plot showing DEGs (*p*-values < 0.05) in the comparison of each NK cell cluster derived from FI^lo^ and FI^hi^ patients. The dot lines show 1.5-fold cutoff. **(D)** GSEA analysis of DEGs in NK cells in the comparison of FI^lo^ and FI^hi^ patients with severe COVID-19. **(E)** UMAP visualization of monocyte clusters. **(F)** Heatmap showing the row-scaled expression of selected signature genes for each cluster. **(G)** GSEA analysis of DEGs in monocytes in the comparison of FI^lo^ and FI^hi^ patients with severe COVID-19. **(H)** Volcano plot showing DEGs (*p*-values < 0.05) in the comparison of each monocyte cluster derived from FI^lo^ and FI^hi^ patients. The dot lines show 1.5-fold cutoff.

Previous studies have reported that NK cells are functionally impaired in severe COVID-19 patients (58, 59). In the study, three NK clusters were identified by transcriptome analysis, including one CD56^bright^ cell (NK3, *NCAM1*^hi^) that expressed an intermediate level of *CXCR4* and high levels of *XCL1* and *AREG*, two CD56^dim^ NK cell clusters with NK1 expressing high levels of *CXCR4*, and pro-inflammatory cytokines *IFNG, TNF, CCL4*, and NK2 expressing high levels of *CX3CR1*, cytotoxicity-related genes, and type I IFN-responsive genes (**Figures 3A, B** and **Figure S3A**). Neither total NK cells nor the three NK clusters were different between FI^hi^ and FI^lo^ groups (p > 0.57) (**Figure S3B**).

Similar to T cells, FI^hi^ patient-derived NK1 cluster (CD56^dim^IFNG^+^) in FI^hi^ patients showed reduced expression of IFN-responsive genes, S100 alarmins, and inhibitory KIR *KIR3DL2*, and increased expression of nuclear receptor NR4A members, *CXCR4, IFNG, CREM*, and *BHLHE40* (**Figure 3C**). The GSEA analysis exhibited that the downregulated genes in FI^hi^ patient-derived NK cells, irrespective of total NK cells or NK clusters, were enriched in interferon signaling, innate immune system, cytokine signaling in immune system, and NK cell-mediated cytotoxicity (**Figure 3D**). An enrichment of cellular responses to external stimuli, regulation of expression of SLITS and ROBOS, and oxidative phosphorylation were observed in the genes upregulated in FI^hi^-derived circulating NK cells (**Figure 3D**).

The circulating monocytes have been shown to be recruited to the lung and play an essential role in the development of pulmonary fibrosis (60, 61). In the study, 4 monocyte clusters were identified in the patients with severe COVID-19 (**Figures 3E, F**, **S3C**). The most prevalent cluster was Mo1 with high expression of pro-inflammatory cytokines such as *IL1B, CXCL8, CXCL2, CCL3*, and growth-regulating molecule *EREG* (**Figure 3F**). Other monocyte clusters included one (Mo2) with high expression of alarmin molecules (S100A8 and S100A9) and lymphoid organ homing molecule (SELL), one (Mo3) with positive expression of IL17RA and STAT3, and one (Mo4, FCGR3A^+^) with high expression of HLA class I and II molecules, and genes involved in inflammation (62, 63), such as *PLAUR, MARCKS, TNF*, and *CCL4* (**Figure 3F**).

Despite the fact that the frequency of monocytes and their clusters was comparable between two groups, panels of gene expression with enrichment of interferon signaling, innate immune system, cytokine signaling in immune system, class I MHC-mediated antigen processing, antigen processing cross-presentation, and neutrophil degranulation were decreased in FI^hi^ patient-derived monocytes (**Figures 3G, H**, **S3D**). Notably, the cells in CXCL8^+^ Mo1 cluster upregulated CSF1R-regulating *EHD1* and activation-related *PROK2* and *MMP19* (**Figure 3H**), suggesting that the activation of these cells may be dysregulated (64, 65). Similar to monocytes, interferon signaling and antigen presentation were enriched in the downregulated genes in FI^hi^ patient-derived dendritic cells and B cells (**Figures S3E**–**H**).

Collectively, these data indicate that global antiviral responses were attenuated in severe COVID-19 patients with high PF index.

### Reduced Lymphoid–Myeloid Cell Interaction in Severe COVID-19 Patients With High PF Index

**Figure 4:**
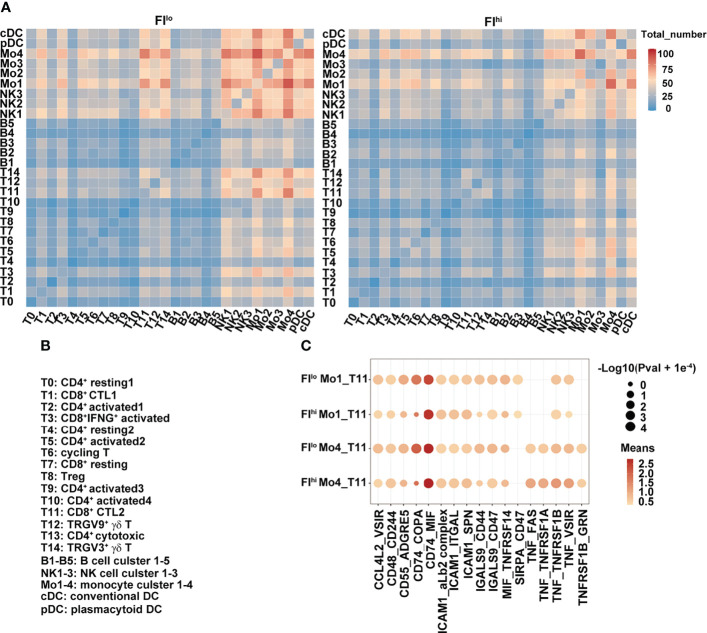
Weakened interaction between T cells and other cell types in FI^hi^ patients with severe COVID-19. **(A)** Heatmap shows the number of ligand and receptor pairs co-expressed among various cell types and cell clusters in the patients with severe COVID-19. The results derived from FI^lo^ and FI^hi^ patients were separately shown. **(B)** Lists of the names of the cell clusters. **(C)** Bubble plot shows mean expression of ligand and receptor pairs co-expressed between monocyte clusters (Mo1 and Mo4) and CTL cluster T11. The results derived from FI^lo^ and FI^hi^ patients were separately shown.

To determine whether the cellular communications were altered in FI^hi^ patients with severe COVID-19, we performed cell–cell interaction analysis among various cell clusters. In FI^lo^ patients, NK cells, monocytes, and DCs closely interacted with each other (**Figures 4A, B**). Except naïve T cells and CD4^+^ activated cells in clusters T9 and T10, CD4^+^ and CD8^+^ activated T cells (T3, T5, T6), Treg cells (T8), and cytotoxic T cells (T1, T11, T12, and T14) all showed interactions with NK cells, monocytes, and DCs. In particular, strong communications were observed between T11/14 and Mo1/Mo4 (Mo1 with high expression of pro-inflammatory cytokines and Mo4 with high expression of HLAs), and between Mo1/4 and other lymphoid and myeloid cells (**Figures 4A, B**). In FI^hi^ patients, however, the overall cell–cell interactions among various cell types were weaker, with a specific and strong reduction in the communication between T cells (T11/T14) and monocytes (Mo1/Mo4) (**Figures 4A, B**). We further examined the ligand and receptor pairs and found that multiple pairs involved in T11 and Mo1/Mo4 interaction were reduced in the FI^hi^ cohort, including CD55-ADGRE5, CD74-COPA, LGALS9-CD47, and MIF-TNFRSF14 (**Figure 4C**). These results indicate that the reduced cytotoxicity found in T cells in the patients with high fibrosis index is partly due to weakened interactions between T cells and monocytes.

### Positive Association of T Cells and PF Index in COVID-19 Patients

**Figure 5:**
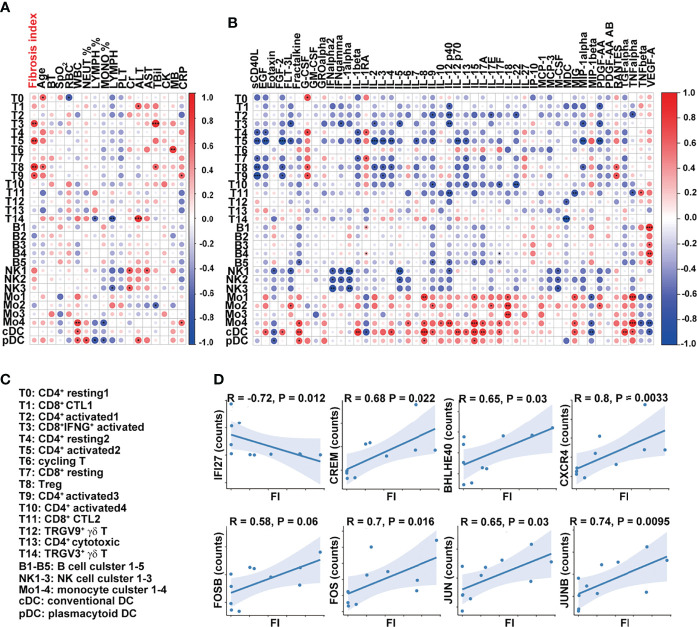
Dysregulated T-cell activation and differentiation in patients with severe COVID-19. **(A, B)** Correlation map reporting Pearson or Spearman correlation values for the comparison of the ratios of various PBMC cell clusters and clinical measures, including FI. **(C)** Lists of the names of the cell clusters. Correlation map reporting Pearson or Spearman correlation values for the comparison of the ratios of various PBMC cell clusters and cytokines. The color of the Correlogram is based on Pearson’s or Spearman’s coefficient values calculated for each couple of measurements in the matrix. Red and blue colors represent positive correlation and negative correlation, respectively. The darker color means the stronger correlation. The following terminology is used to denote the statistical significance: *p < 0.05, **p < 0.01, ***p < 0.005; ns, not statistically significant. **(D)** Correlation analysis of FI and the expression levels of indicated genes in T cells. The inflammation-related genes were selected in the analysis and the ones with significant correlation with FI were shown. Sample 07 was not included in the correlation analysis as its fibrosis index was 0.7, much higher than the rest of the samples (<0.2).

We next determined whether the transcriptome features of PBMCs were related to FI or cytokines in the patients with severe COVID-19. The percentages of effector T-cell clusters, T3, T5, T8, and T9 were positively correlated with FI values in the patients (T3, T5, and T8: *p* < 0.01; T9: *p* < 0.05). Although other cell types or clusters were not associated with FI values, the ratios of FCGR3A^+^HLA^hi^ monocyte (Mo4), cDCs, and pDCs were positively correlated with white blood cell counts (Mo4: *p* < 0.01; cDCs, pDCs: *p* < 0.05) while the percentages of Mo4 cells and pDCs were negatively correlated with circulating monocyte ratios (*p* < 0.05) (**Figure 5A**). Notably, the percentages of T14/T1 were positively associated with serum ALT level (T14: *p* < 0.001; T1: *p* < 0.05) while that of NK1 was positively associated with AST and Cr levels (*p* < 0.05). The close interaction between T cells and monocytes suggests that activated T cells may contribute to pulmonary fibrosis while terminally differentiated cytotoxic T cells may contribute to the liver damage in the patients with severe COVID-19.

The analysis of the cell clusters and cytokines also revealed that sCD40L was negatively associated with the ratios of CD4^+^ and CD8^+^ naïve and activated T-cell clusters (T4, T7, T5, T8, and T9) while the percentage of cluster T5 (CD4^+^ activated T-cell cluster) was negatively associated with a variety of growth factors and cytokines, including IL-2, IL-3, IL-4, IL-7, IL-9, IL-12 p70, IL-13, IL-1β, MIP-1α, FLT-3L, EGF, FGF-2, and PDGF-AA (**Figure 5B**). The cells in clusters cDC and Mo4 were similar in their positive associations with inflammatory cytokines, including fractalkine, IL-8, IL-12, IL-15, IL-17A, IL-17F, MIG, and TNFα (**Figures 5B, C**).

At the transcription level, the expressions of interferon-responsive gene *IFI27* in T cells were negatively correlated with FI values while those of inflammatory cytokine-regulating TFs (*CREM, BHLHE40*), chemokine receptor *CXCR4*, and AP-1-related molecules (*FOSB, FOS, JUNB*, and *JUN*) in T cells were positively correlated with FI values (**Figure 5D**). Together, these results suggest that T-cell activation and differentiation were dysregulated in the patients with severe COVID-19. CREM^hi^BHLHE40^hi^ T cells in these patients upon admission may contribute to the development of pulmonary fibrosis at a later point.

## Discussion

Resulting from severe inflammatory and epithelial cell damage, some COVID-19 patients developed PF, which remained in the convalescent stage. To investigate how immune responses influenced pulmonary fibrosis progression, we performed scRNA-seq using PBMCs isolated from the convalescent patients 14 days after discharge. The main findings of this study include the following: (1) the frequencies of CD3^+^ T cells, activated CD4^+^ T cells, and Treg cells were significantly higher in FI^hi^ COVID-19 patients compared with FI^lo^ subjects; (2) DEGs and GESA analysis identified downregulation of IFN-responsive genes as well as impaired IFN signaling pathways in dysregulated T cells and NK cells; and (3) the expression of IFN-responsive genes and S100A8 was negatively associated with FI values among 12 patients.

Many peripheral soluble and cellular factors have been found to be associated with the disease severity of COVID-19 infection, such as reduced type I interferon and impaired IFN responses (21, 66–68), elevated NF-κB-dependent pro-inflammatory cytokines (20, 26, 69–73), decreased lymphocytes (20, 27–29), downregulated MHC class II expression in monocytes (20, 24, 30–34), and CD4^+^ activation and differentiation (35, 36). Whether these severity-related factors are also associated with PF is not clear. We compared the clinical measures, cytokines, and scRNA transcriptome of PBMCs in 12 patients with severe COVID-19 upon admission and analyzed their association with PF within 14 days of discharge. We found that the transcriptome of almost all the cell types in PBMCs in the patients with high level of CT imaging-based FI had decreased expression of genes enriched in IFN responsiveness, innate immune responses, antigen presentation, and cytotoxicity. The reduction in circulating cytokines that promote T-cell survival and growth substantially decreased interaction between cytotoxic T cells and monocytes, and an expansion of T cells. In particular, CD4^+^ T-cell clusters with the transcriptome features of high levels of endoplasmic reticulum (ER) stress and inflammation-related TFs were associated with severe COVID-19 patients with high level of FI.

Type I IFN produced by T and non-T cells and IFN responsiveness within T cells are critical in regulating T-cell survival, proliferation, differentiation, and antiviral response (74, 75). Optimal activation and differentiation of T cells also require close and dynamic interactions with antigen-presenting cells (APCs) such as DCs, macrophages, and B cells. The negative association of T cell-derived IFN-responsive gene expression with FI and the substantially reduced T monocyte interaction in the FI^hi^ cohort further suggest that T-cell differentiation and function are dysregulated, likely leading to unsuccessful control of virus and continuous production of inflammatory cytokines.

Within the 12 patients with severe COVID-19, the proportions of circulating NK cells, B cells, myeloid cells, or their cell clusters were not significantly different between FI^hi^ and FI^lo^ cohorts or significantly associate with FI values. However, we found a general decrease in the expression of genes related to IFN responses, innate immune responses, antigen presentation, and cytotoxicity in the patient cohort with high level of FI. Although these changes are consistent with the severity of the disease, it is somewhat contradictory to the findings of amplified type I IFN response in monocytes or lung cells in idiopathic pulmonary fibrosis (76, 77). Whether this is due to different pathogenesis between viral and non-viral infection-induced PF is not clear. The contribution of these PBMCs with low IFN responsiveness and high CXCR4 expression to the development of PF also awaits further investigation. Nevertheless, the more severe defects in IFN responsiveness in PBMCs in FI^hi^ patients suggest that these patients are disabled in effective control of virus and such impaired viral control may contribute to dysregulated immune cell differentiation and inflammation that eventually leads to fibrosis.

The incidence of pulmonary fibrosis by COVID can be estimated based on a 15-year observational study of lung pathology after SARS. Despite the fact that most SARS patients recovered without any sign of lung damage, pulmonary fibrosis could still be detected in 20% after 5–10 years of discharge. Based on these data, the incidence rate of post-COVID lung fibrosis could be approximately about 2%–6% over the course of the pandemic (78). To some extent, the variants of SARS-CoV-2 have different mutations. However, they do share the same infection mechanism *via* binding of Spike protein to ACE2 on host cells. Therefore, the findings in the study could be attributed to the severe infection among all variants.

The current study has some limitations. First, the sample size was small with only 12 patients included, making the statistical analysis less powerful. Second, the serum and PBMC samples were collected only once upon admission. It will provide more information in the development of PF if a longitudinal study is applied.

Taken together, transcriptome analysis of PBMCs at the single-cell level, serum cytokines, and other clinical measures in the patients with severe COVID-19 revealed an association of PF with increased pathogenic Th17 cells and generally dampened IFN responses.

## Data Availability Statement

The datasets presented in this study can be found in online repositories. The names of the repository/repositories and accession number(s) can be found at: https://www.ncbi.nlm.nih.gov/, accession ID: GSE165182.

## Ethics Statement

The studies involving human participants were reviewed and approved by the Institutional Review Board of the Capital Medical University. The patients/participants provided their written informed consent to participate in this study.

## Author Contributions

YF and QG designed the research and wrote the manuscript. ZW, YZhang, and RY performed the research and analyzed the data. YW, JG, RS, YZhou, and LS provided critical help in data analysis. LS edited the manuscript and provided critical opinions. All authors contributed to the article and approved the submitted version.

## Funding

This work was supported by grants from the National Natural Science Foundation of China (32070897 and 31872734, QG; 82070627, YZ), the Foundation for Innovative Research Groups of the National Natural Science Foundation of China (81621001, QG), Beijing Natural Science Foundation (7202079, QG), the Non-Profit Central Research Institute Fund of Chinese Academy of Medical Sciences, 2018PT31039, the Open Funding Project of National Key Laboratory of Human Factors Engineering (6142222200105, QG), Beijing Science Foundation of China (Z201100001020004, Z201100005520062, and Z201100007920017, YF), Beijing Municipal Institute of Public Medical Research Development and Reform Pilot Project (2016-2, YF; 2021-10, YZ), and Beijing Municipal Natural Science Foundation (7222090, YZ).

## Conflict of Interest

The authors declare that the research was conducted in the absence of any commercial or financial relationships that could be construed as a potential conflict of interest.

## Publisher’s Note

All claims expressed in this article are solely those of the authors and do not necessarily represent those of their affiliated organizations, or those of the publisher, the editors and the reviewers. Any product that may be evaluated in this article, or claim that may be made by its manufacturer, is not guaranteed or endorsed by the publisher.
