# Supplementary material for: Landscape of Peripheral Blood Mononuclear Cells and Soluble Factors in Severe COVID-19 Patients With Pulmonary Fibrosis Development
Source: Front Immunol. 2022 Apr 26;13:831194. doi: 10.3389/fimmu.2022.831194 (PMC9088015; doi:10.3389/fimmu.2022.831194)
Supplement: Supplementary file 1 [file DataSheet_1.docx]

**Supplementary materials**

**Landscape of peripheral blood mononuclear cells and soluble factors in severe COVID-19 patients with pulmonary fibrosis development**

Running title: scRNA-seq of COVID-19 patients with pulmonary fibrosis

Zhuolin Wang^1†^, Yang Zhang^2,3†^, Rirong Yang^4,5†^, Yujia Wang^1^, Jiapei Guo^1^, Ruya Sun^6^, Yuan Zhou^6^, Li Su^7^*, Qing Ge^1,8^*, Yinmei Feng^2^*

^†^These authors have contributed equally to this work and share first authorship

^*^To whom correspondence may be addressed:

Yingmei Feng, Beijing Youan Hospital, Capital Medical University, Beijing, 100069, China. Email: [yingmeif13@sina.com](mailto:yingmeif13@sina.com);

Qing Ge, Peking University, 100191. Email: [qingge@bjmu.edu.cn](mailto:qingge@bjmu.edu.cn);

Li Su, Peking University, Beijing 100191, China, Email: [dudu.su@163.com](mailto:dudu.su@163.com)

**Table S1. DEGs in the comparison of each T cell clusters derived from FI^lo^ and FI^hi^ patients**

| T clusters | Genes | Avg_Log2FC | -Log10 (*P* value) |
| --- | --- | --- | --- |
| T1 | NR4A2 | 1.120 | 1.510 |
|  | IFNG | 1.069 | 1.703 |
|  | BHLHE40 | 0.831 | 1.366 |
|  | CXCR4 | 0.807 | 1.324 |
|  | NLRP1 | 0.772 | 2.062 |
|  | JUN | 0.749 | 1.363 |
|  | S100A11 | -0.636 | 1.723 |
|  | PRF1 | -0.873 | 1.398 |
|  | IFITM1 | -0.922 | 2.307 |
|  | PLAC8 | -0.957 | 1.987 |
|  | IFITM3 | -1.896 | 3.762 |
|  | OAS1 | -2.219 | 3.220 |
| T3 | CREM | 1.198 | 3.207 |
|  | TNF | 1.075 | 2.059 |
|  | NR4A2 | 1.005 | 4.017 |
|  | CD69 | 0.916 | 4.017 |
|  | REL | 0.679 | 2.293 |
|  | FOS | 0.780 | 2.004 |
|  | S100A11 | -0.729 | 1.879 |
|  | PRF1 | -0.851 | 1.403 |
|  | IFITM1 | -0.877 | 2.118 |
|  | IFI35 | -1.096 | 1.320 |
|  | IFITM3 | -1.873 | 2.118 |
| T5 | PER1 | 0.973 | 1.462 |
|  | NR4A2 | 0.958 | 4.375 |
|  | TNFAIP3 | 0.890 | 2.576 |
|  | PTGER4 | 0.798 | 3.041 |
|  | SLC2A3 | 0.758 | 2.576 |
|  | GLRX | -0.602 | 1.357 |
|  | IFITM1 | -0.734 | 1.353 |
|  | PLAC8 | -0.815 | 1.670 |
|  | STAT1 | -1.277 | 1.715 |
| T8 | PER1 | 1.197 | 1.720 |
|  | CXCR4 | 1.002 | 2.079 |
|  | NR4A2 | 0.957 | 2.340 |
|  | TNFAIP3 | 0.836 | 2.577 |
|  | ICOS | 0.809 | 2.079 |
|  | IFITM1 | -0.815 | 1.612 |
|  | STAT1 | -0.926 | 1.527 |
|  | IFI35 | -1.049 | 1.477 |
|  | IFI44 | -1.174 | 1.416 |
|  | MX2 | -1.309 | 1.655 |
| T9 | CREM | 1.667 | 2.194 |
|  | PDE4B | 1.353 | 1.999 |
|  | SLC2A3 | 1.169 | 1.595 |
|  | NR4A2 | 1.033 | 1.662 |
|  | BST2 | -2.010 | 1.361 |
|  | IFITM3 | -2.315 | 2.488 |

DEGs were calculated by DESeq2.


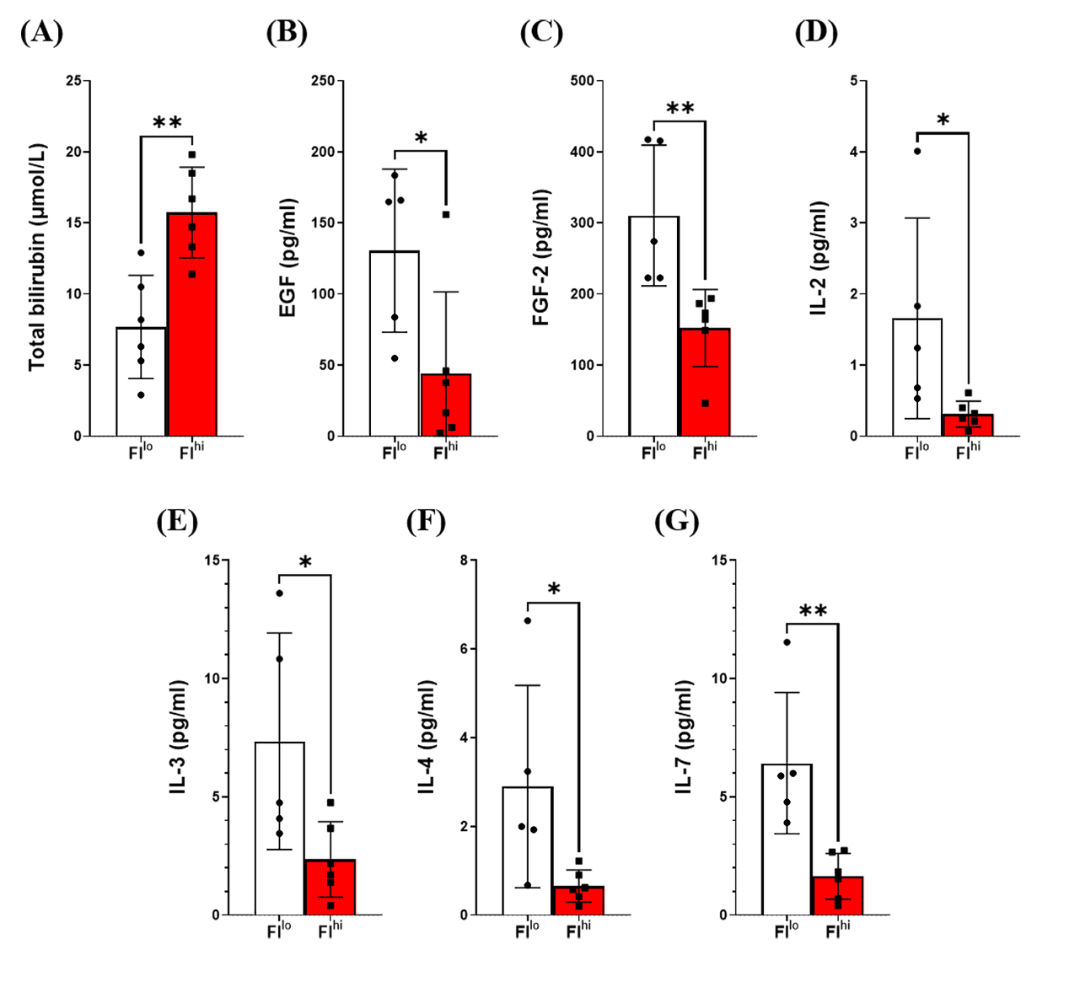


**Fig. S1. Serum levels of total bilirubin (A), EGF (B), FGF-2 (C), IL-2 (D), IL-3 (E), IL-4 (F), IL-7 (G) measurement in FI^lo^ and FI^hi^ patients.** Data are shown as means± sd. Statistically significant differences between samples groups were estimated by GraphPad Prism software (Version 9.0) using Student’s t-test. *: p < 0.05; **: p < 0.01.


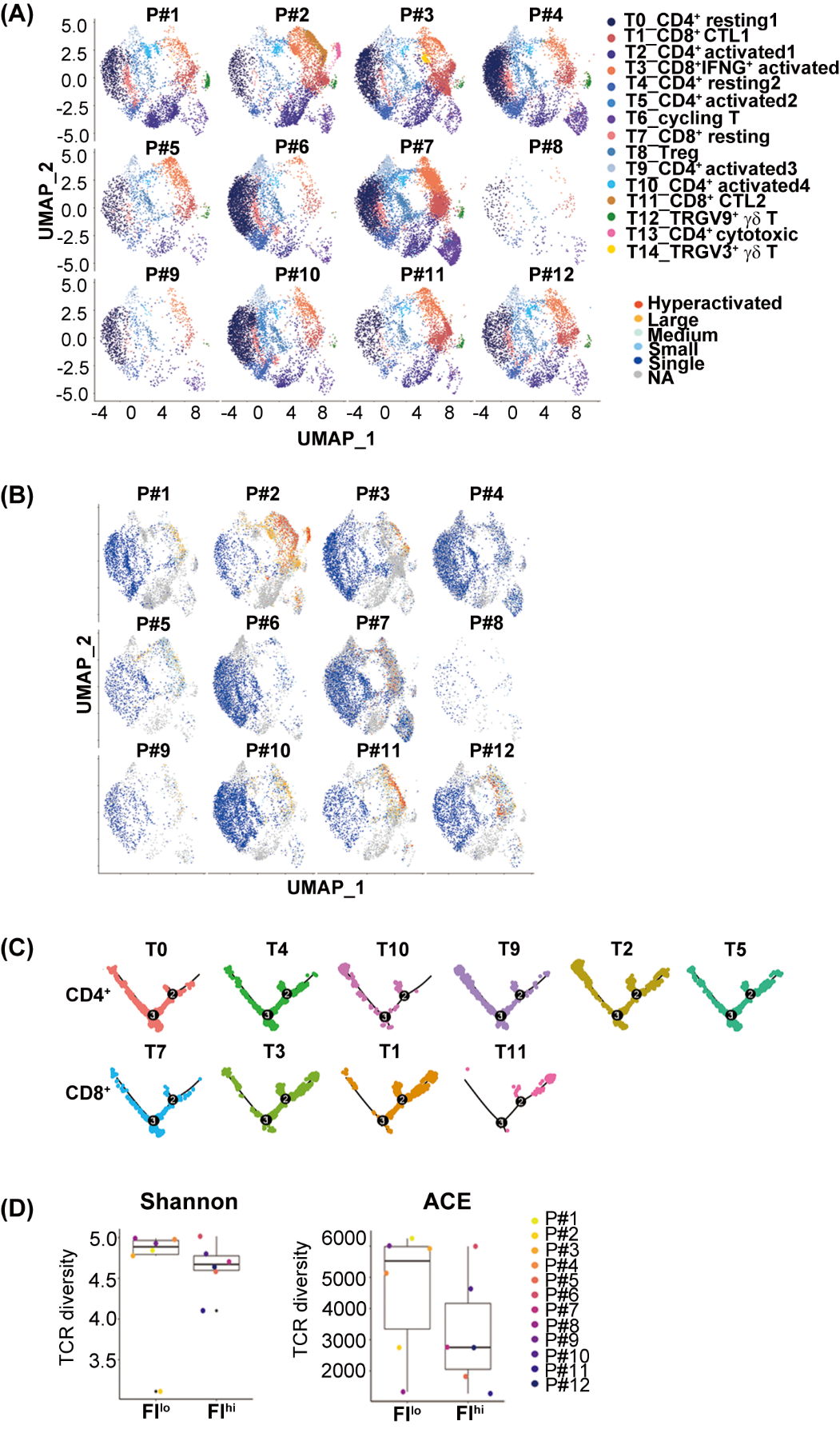


**Fig. S2. T cell composition in the patients with severe COVID-19.** (A). UMAP visualization of T cell clusters in individual patients. (B). Distribution of single to hyperactivated clonotypes in individual patients. (C). Monocle trajectories of T cells colored by cluster identity. (D). Comparison of TCR diversity between FI^lo^ and FI^hi^ patients by Shannon and ACE analysis.


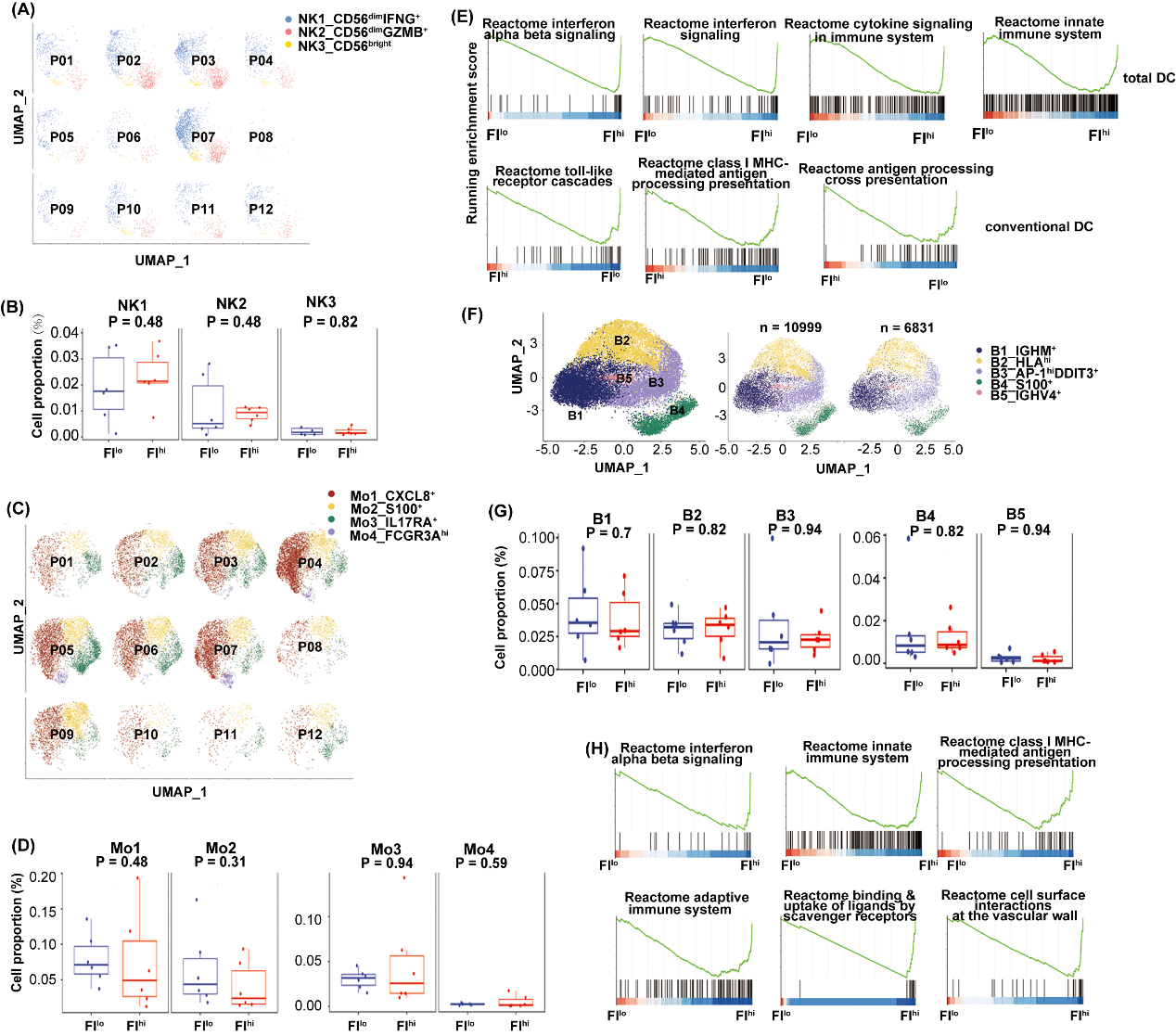


**Fig. S3. NK cell and monocyte composition in the patients with severe COVID-19.** (A). UMAP visualization of NK cell clusters in individual patients. (B). Comparison of the proportion of NK cell clusters between FI^lo^ and FI^hi^ patients. (C). UMAP visualization of monocyte clusters in individual patients. (D). Comparison of the proportion of monocyte clusters between FI^lo^ and FI^hi^ patients. (E). GSEA analysis of DEGs in total DCs and cDCs in the comparison of FI^lo^ and FI^hi^ patients with severe COVID-19. (F). UMAP visualization of B cell clusters. (G). Comparison of the proportion of B cell clusters between FI^lo^ and FI^hi^ patients. (H). GSEA analysis of DEGs in B cells in the comparison of FI^lo^ and FI^hi^ patients with severe COVID-19.
